# Visceral pressure stimulator for exploring hollow organ pain: a pilot study

**DOI:** 10.1186/s12938-021-00870-y

**Published:** 2021-03-25

**Authors:** Michael DeLong, Mauricio Gil-Silva, Veronica Minsu Hong, Olivia Babyok, Benedict J. Kolber

**Affiliations:** 1 Center for Advanced Pain Studies, Department of Neuroscience, University of Texas at Dallas, 800 W. Campbell Rd., Richardson, TX 75080 USA; 2 Department of Biological Sciences, Duquesne University, 600 Forbes Avenue, Pittsburgh, PA 15217 USA

**Keywords:** Bladder pain, Timed pressure regulator, Visceral pain, Colorectal pain

## Abstract

**Background:**

The regulation and control of pressure stimuli is useful for many studies of pain and nociception especially those in the visceral pain field. In many in vivo experiments, distinct air and liquid stimuli at varying pressures are delivered to hollow organs such as the bladder, vagina, and colon. These stimuli are coupled with behavioral, molecular, or physiological read-outs of the response to the stimulus. Care must be taken to deliver precise timed stimuli during experimentation. For example, stimuli signals can be used online to precisely time-lock the stimulus with a physiological output. Such precision requires the development of specialized hardware to control the stimulus (e.g., air) while providing a precise read-out of pressure and stimulus signal markers.

**Methods:**

In this study, we designed a timed pressure regulator [termed visceral pressure stimulator (VPS)] to control air flow, measure pressure (in mmHg), and send stimuli markers to online software. The device was built using a simple circuit and primarily off-the-shelf parts. A separate custom inline analog-to-digital pressure converter was used to validate the real pressure output of the VPS.

**Results:**

Using commercial physiological software (Spike2, CED), we were able to measure mouse bladder pressure continuously during delivery of unique air stimulus trials in a mouse while simultaneously recording an electromyogram (EMG) of the overlying abdominal muscles.

**Conclusions:**

This device will be useful for those who need to (1) deliver distinct pressure stimuli while (2) measuring the pressure in real-time and (3) monitoring stimulus on–off using physiological software.

**Supplementary Information:**

The online version contains supplementary material available at 10.1186/s12938-021-00870-y.

## Background

Visceral pain includes acute and chronic pain associated with the viscera (e.g., internal organs). There are a number of unique characteristics of visceral pain that set it apart from somatic pain including unique sets of nociceptors, the presence of silent nociceptive neurons, and the phenomenon of referred pain [1, 2]. Chronic visceral pain conditions include irritable bowel syndrome, chronic prostatitis, and chronic pelvic pain syndromes. The economic impact varies across the conditions, but can reach up 1.35 billion US dollars per year [3]. Five percent of women are thought to be at risk of chronic pelvic pain, and chronic prostatitis affects 4.5–9% of men in the United States [4, 5]. These disorders are difficult to treat and many have unknown etiology. Animals (e.g., mice, rats, cats) provide critical models to understand and ultimately treat chronic visceral pain conditions. Numerous challenges exist in the study of visceral pain in animals. One of the biggest challenges is the method used to stimulate the visceral organ. Unlike somatic structures that can be easily accessed from the body surface, visceral structures must be stimulated with internal methods.

For vaginal, bladder, and rectal studies, the most common method involves inserting a catheter or balloon into the organ. This catheter or balloon is then connected to an air tank or pump to deliver air or liquid (e.g., water or saline) to the organ. Simultaneously, researchers measure the physiological, behavioral, and molecular impacts of this stimulation. For example, in the urinary bladder distention (UBD) visceromotor response (VMR) model, a mouse or rat’s bladder is catheterized (typically female animals only) through the urethra [6, 7]. Distensions with graded air pressure (from 15 to 75 mmHg) induce corresponding graded reflexive contractions of the overlying abdominal muscles. These contractions can be quantified using an electromyogram (EMG) and are used as an indirect measure of pain-like responses to the stimulus. In human volunteers, similar stimuli induce graded feelings of discomfort and pain [8]. That is, low pressures are only mildly discomfortable while high pressures are described as painful. A necessary component of such studies is the ability to accurately measure pressure, closely control stimulus on/off, and time-lock stimuli to physiological measurements (e.g., VMR). A number of devices have been designed over the years with the first publication of such a remotely controlled device in 1987 [9]. The current manuscript describes a modern re-design of this device using off-the shelf equipment to reliably control stimuli pressure on/off gating, accurately measure pressure during stimulation, and provide stimulus markers for online time-locking of stimuli to physiological measures. The device was used to measure pain-like responses to bladder distention in an anesthetized mouse.

## Results

### VPS build

**Fig. 1:**
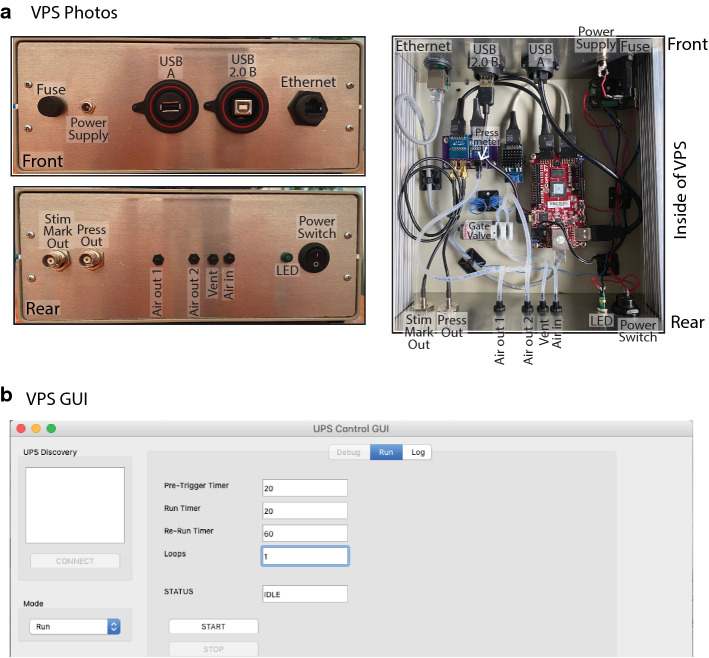
Photo montage of VPS and GUI for VPS control. **a** Photos showing front, rear, and inside of VPS device (with individual parts labeled). **b** Screen shot of GUI for VPS control. Controls allow the user to set the pre-trial interval (set to 20 s in example), the length of an actual pressure trial (set to 20 s in example), the length of the intertrial interval (set to 60 s in example), and the number of trials to be run (“loops”)

**Fig. 2:**
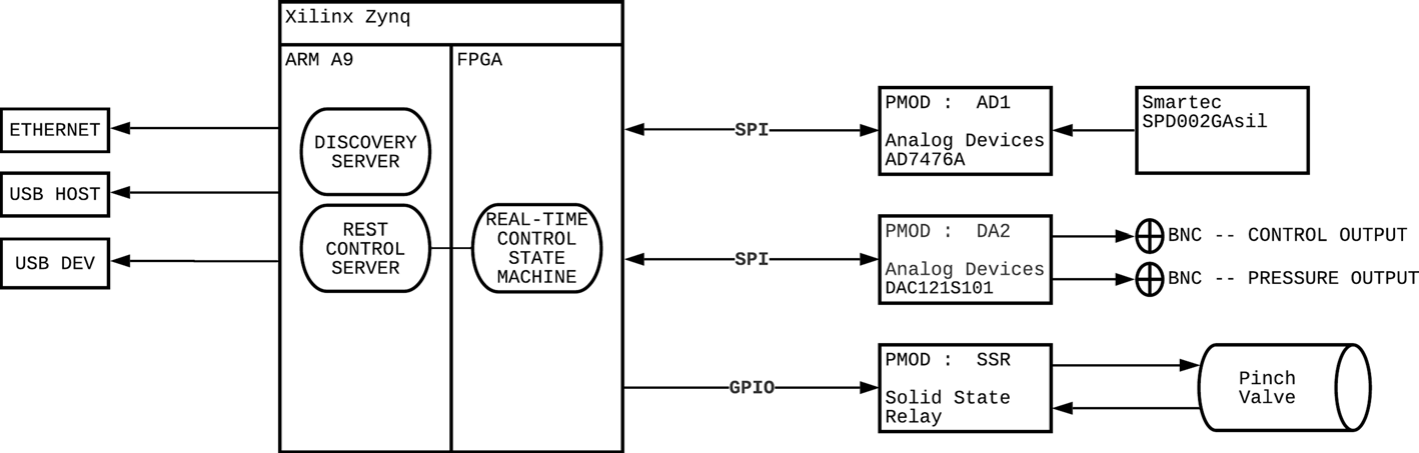
Circuit diagram for VPS build

In this experiment, we sought to build a modern pressure regulator that would allow us to easily control pressure stimuli for use in animal models of visceral pain. We designed the system, the Visceral Pressure Stimulator (VPS; Fig. 1a) to have a pinch valve to gate the air stimulus, an analog-to-digital pressure sensor to accurately read the pressure when the pinch valve was “open”, and a stimulus marker output function. The stimulus marker function included a marker at the pre-distention start and during the entire pinch valve open time period (Fig. 4a). The system is fully controllable via the standard representational state transfer (REST) web protocol. We included a prototype graphical user interface (GUI) based on Python3 and PyQt5 which exposes the REST-based control to a simple GUI. The GUI (Fig. 1b) allows the researcher to control the length of the pre-distention interval, the distention trial length and the intertrial interval. The system can also be set to run in loops for multiple trials in a row. The circuit diagram for the build can be seen in Fig. 2. The VPS was designed and built using off-the-shelf components and open-source software. The primary processor board in the system is a Digilent Cora Z7 which features the Xilinx Zynq 7000 processor. This board allows for connections to Arduino shields and Pmod boards in addition to standard PC interfaces. The Xilinx Zynq part has a die coupled dual core ARM A9 processor and field-programmable gate array (FPGA). The VPS uses a full custom embedded Linux distribution which facilitates standard Ethernet and USB external connections that are utilized in the VPS. The FPGA is programmed to handle the real-time aspects of the system including the analog-to-digital (ADC) and digital-to-analog (DAC) for air pressure measurement, the BNC based voltage outputs that interface to the larger system and the pinch valve. Standard Pmod boards were used for ADC, DAC, and pinch valve control with minor modifications for packaging.

### Testing of VPS in a mouse model of bladder pain

**Fig. 3:**
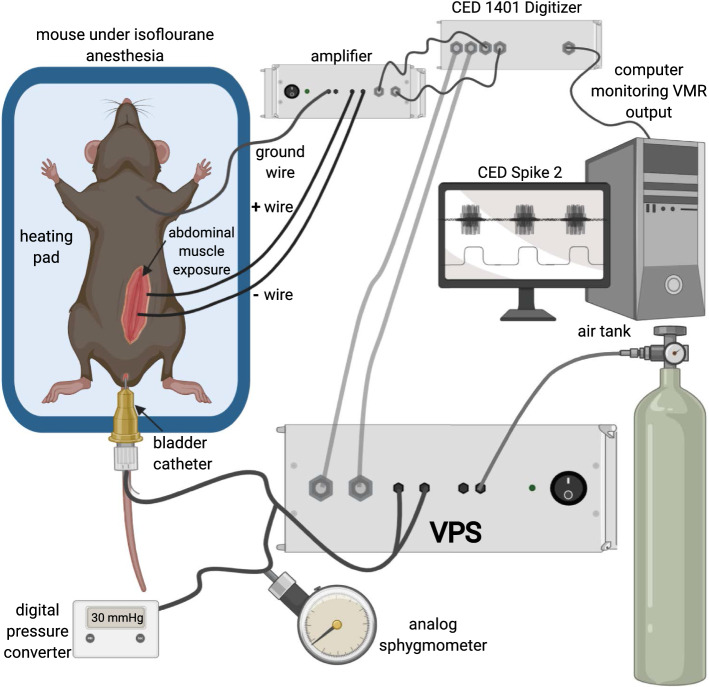
Schematic diagram for VPS testing with UBD-VMR method in mice. Illustration of UBD-VMR setup with schematic for testing VPS showing air source, VPS, secondary pressure measuring devices, and ADC (1401 Plus)

**Fig. 4:**
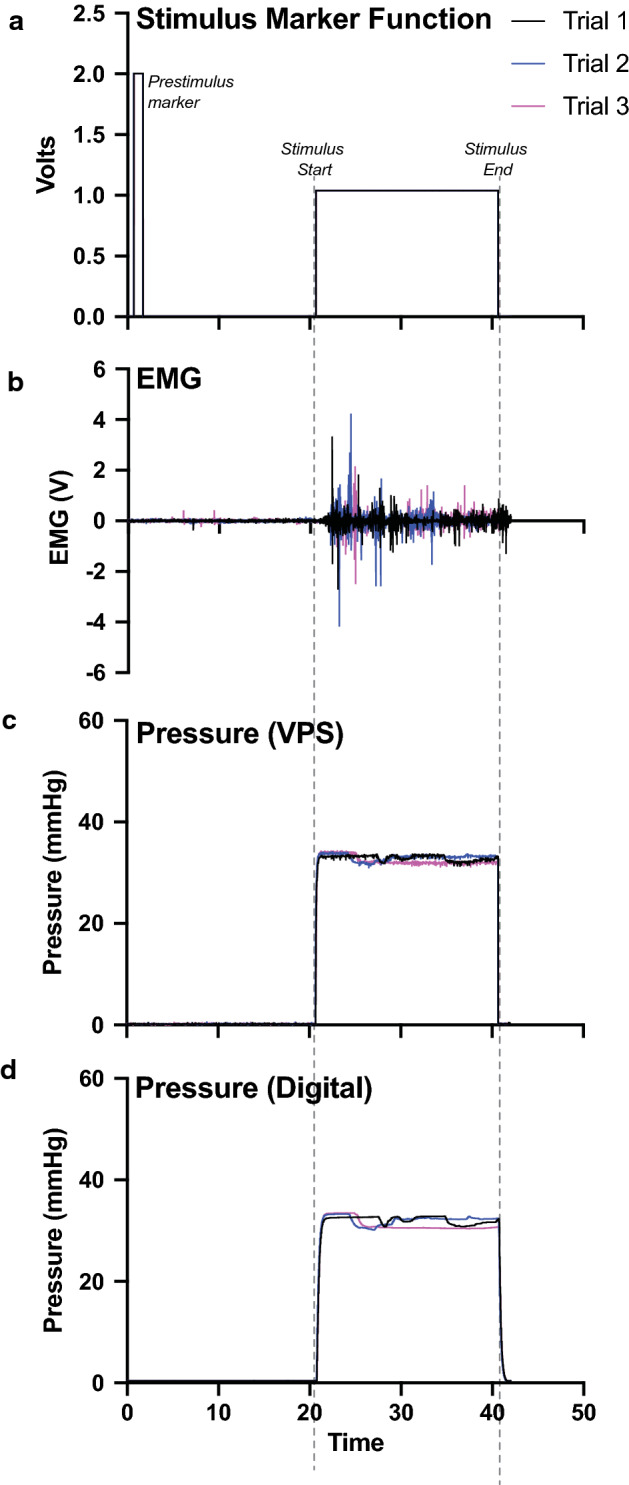
Demonstration of three UBD-VMR trials of 20 s at 30 mmHg. All trials were controlled with the VPS GUI. **a** Plot is the stimulus marker function (from VPS) showing 2 V pre-stimulus marker along with 1 V stimulus marker. **b** Plot of raw EMG (volts) showing bladder distention evoked increase in EMG signal after stimulus start and tapering off of signal at stimulus end. **c** Plot of pressure (mmHg) read from VPS. **d** Plot of pressure read from inline analog-to-pressure converter. Initial pressure setting was set using an analog sphygmometer to be at “30 mmHg” connected to an analog air valve. Pressure setting was not adjusted between trials. Plots were matched to pre-stimulus marker

To evaluate the potential of the VPS for visceral nociception testing, we utilized the mouse UBD-VMR procedure (Fig. 3). In this model, we catheterized the bladder of an anesthetized female mouse. The catheter is connected to the VPS allowing discrete stimuli to be applied to the bladder. The goal of this demonstration experiment was to show the utility of the VPS interface in directing different stimulus parameters to the mouse. We tested 20 s 30 mmHg pressure trials using the VPS with an intertrial interval of 60 s and three loops. The initial air pressure was set using an analog sphygmometer upstream of the VPS. The pressure was set to “30 mmHg”. As can be seen in Table 3, the actual pressure read was ~ 31 mmHg which is in manufacturer’s tolerance range of the upstream analog sphygmometer (± 3 mmHg). Pressure was measured using three different methods. This included a second analog commercial sphygmometer downstream of the VPS, a custom inline analog-to-digital pressure converter downstream of the VPS, and the pressure sensor in the VPS. Spike 2 was used to record (1) the stimulus markers from the VPS (Fig. 4a) (2) the electromyogram (EMG) of the abdominal muscles overlying the bladder (Fig. 4b) (3) pressure from the VPS (Fig. 4c), and (4) pressure from an additional custom analog-to-digital pressure converter (Fig. 4d). The stimulus marker function included two markers. The first is a 2 V signal that begins at the start of each pre-distention trial and lasts for 1 s. The second 1 V marker begins 20 s after the pre-distention marker and lasts for 20 s in these distention trials. In other words, the 1 V signal is a marker indicating when the pressure pinch valve on the VPS (see Fig. 1a) is open. The EMG to bladder distention occurs shortly following stimulus start as pressure increases. There is a slight delay in the EMG response due to (1) the time it takes for air to fill the tubing and urinary bladder once the VPS pinch valve opens (~ 1 s) and (2) the time it takes for the in vivo spinal reflex system to register the distention and induce muscle contractions (~ 1 s). The total delay is actually variable between mice since it includes both the standard time for air to reach the bladder and the biological response of the animal to the stimulus (i.e., bladder distended, sensory neuron activated, spinal cord interneuron activated, efferent reflex initiated). For the demonstration trials, the delay was ~ 2 s total. Similarly, as the custom converter is downstream of the VPS controller, there is a slight lag in the registered pressure both at stimulus on and stimulus off. The analog sphygmometer was manually observed and the pressure was noted half way through each trial. There was strong correlation between the VPS pressure sensor and the custom converter (Table 3). Following testing, a custom script in Matlab was used to convert the raw EMG signal into a normalized visceromotor response (VMR) during each distention trial; the script also calculates the average pressure during each trial (Table 3).

**Fig. 5:**
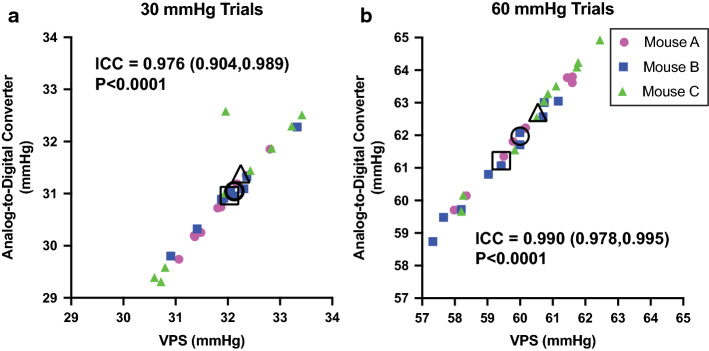
Real-world comparison of pressure measured from VPS versus separate pressure meter. Data are presented from three different female mice (mice A, B, C). To compare the inter-rater reliability between the VPS pressure transducer and a separate pressure transducer in our analog-to-digital converter, data from multiple trials **a** at 30 mmHg or **b** 60 mmHg are plotted against each other for the two transducers. ICC (95% confidence intervals) and *P* values are provided for each graph. There is excellent reliability between the two transducers. Variability between trials within mice is due to variability in the manual adjustment of pressure that was done as trials alternated between 30 and 60 mmHg. Pressure target during manual adjustment was completed using a separate analog sphygmometer with a ± 3 mmHg error. Large open symbols show mean pressure for that particular animal across all 10 trials

Following the initial development of the VPS, we initiated a period of real-world usage of the machine in the laboratory. Over the course of 2 months, we tested 18 female mice utilizing pressures ranging from 15 to 75 mmHg depending on the specific experimental parameter. Across these studies, we completed 1200 total trials using both Macintosh- and Windows-based computers to run the VPS. To assess whether the pressure of the VPS ran true to the pressure detected by our analog-to-digital converter, we extracted 20 trials from three separate mice. Pressure recordings from the VPS and analog-to-digital converter were analyzed for intra-rater reliability. The intra-class correlation coefficient (ICC) between 30 mmHg pressure readings was 0.98 (*P* < 0.0001) and the ICC between 60 mmHg pressure readings was 0.99 (*P* < 0.0001) suggesting excellent reliability (ICC > 0.9) of pressure readings across multiple trials and testing days (i.e., different animals) (Fig. 5).

## Discussion and conclusions

Visceral pain is challenging to treat, in part, due to the lack of known mechanisms for human disease. In order to investigate these mechanisms, it is necessary to study visceral nociception in animal models. For hollow organ studies (e.g., bladder, vagina, rectum), the ability to precisely deliver pressure stimuli is critical. In this experiment, we designed a modern pressure regulator, the visceral pressure stimulator (VPS), to control pressure stimuli. This device was built using off-the-shelf parts and a Linux-based control system to facilitate rapid adoption in the research community. We built the system to be controlled via standard web protocols and the included example GUI. The GUI allows the researcher to quickly change the length of the pre-distention trial, the length of the actual distention trial, and the intertrial interval. By including a loop option, the system also allows a researcher to run multiple trials back-to-back. In addition, the control of the VPS instrument is automated using REST-based programmatic control, facilitating fully automated data collection systems. The inclusion of a stimulus marker output from the VPS allows for time-locking of the pressure stimulus to the EMG recorded in this experiment. However, this marker could easily be used for other physiological measurements (e.g., neuronal activity) and even behavior (e.g., locomotor activity captured with a video system). The system is also versatile and could easily include additional options including a trigger option and could be adapted to a fluid pump for delivery of liquid pressure stimuli. Python is the primary development language for the VPS system on both the user interface and on the embedded Linux system. This type of high-level and flexible language allows for quick changes and experimentation. Being cross-platform, the GUI works on Windows, Linux and Macintosh-based computers. Additionally, the fully featured embedded Linux system running on the VPS can support a vast array of programming languages from very low level (C/C++/Rust) to high-level application-based languages (MATLAB scripts via Octave) and everything in between.

This device provides an important modern update to the last publication of a pressure controller from 1987 [9]. That device used technology which necessitated multiple additional control devices all of which are subject to failure. Interestingly, there are certain elements of the original design that are still used. These, perhaps not surprisingly, are the ADC’s used to measure air pressure and control the pinch valves. This technology has not significantly changed in the last 30 years. However, what has changed dramatically is the tremendous growth in the microcircuitry field. This growth allows for a significant amount of miniaturization in the present build. This miniaturization translates into cost savings for assembly time. What might previously have taken many tens of hours to build now takes less than an hour to assemble. Given that many older capacitors and switches are outdated, they can also be difficult to find if one fails. The current build uses mainly commercially available parts that are easily obtained providing a significant benefit in the time needed to procure parts.

To our knowledge, there are no commercially available units to control and measure pressure while including a stimulus marker for hollow organ distention experimentation. There are units to measure pressure of course (e.g., World Precision Instruments Model II-MRBP-M) that have been used in hollow organ pain testing in rodents [10, 11] but these units do not control air/liquid flow nor do they send stimulus markers for time-locking of experimental trials. Similarly, there are units designed to measure bladder function using cystometry (e.g., Med Associates Cystometry Station of Mouse Model CAT-CYT-M), but again, this is for measurement of bladder function not induction of pressure stimuli [12].

While there are no commercial comparisons to the VPS, there are reports in the literature of other custom units [13–15] or methods of pressure reading and recording [16, 17]. To our knowledge, none of these other units are described in detail preventing a clear comparison of advantages and disadvantages to the VPS. We can however speak to the older custom machine (Washington University School of Medicine Electronics Shop) that our research group has used in the past [6, 7, 18, 19]. This self-contained unit relies on older Omron timers and rotary switches to select the taps to monitor the decade monitors. Push buttons (start, stop, pause, and reset) send a digital level signal to the various logic inputs to the panel and the internal digital logic to control the progression of trial sequences. The pressure pinch valve and transducer are equivalent to the VPS unit. While functionally similar to the VPS presented here, the older unit is difficult to troubleshoot. It also necessitates a considerable amount of manual wiring to build. This makes each unit significantly more time-consuming and costly to manufacture. Since the hardware is static and there is no software, the system is not as flexible for adapting to different experimental setups and requirements as the new VPS is with its off-the-shelf but modifiable circuitry and multiple operating system compatible software. An advantage of the older unit is that it is self-contained and does not require a separate computer to function. However, a computer is needed anyway to receive the signals from both models so that “disadvantage” of the VPS is negligible.

Overall, in this experiment we have described a valuable modern update to an important instrument used by biomedical researchers in the visceral pain field. This device has been designed for flexibility in use and is especially useful in conjunction with physiological experiments where time-locking of stimuli with pain measures is necessary. To ensure that other laboratories could duplicate this device, we have included the circuit diagram (Fig. 2), parts lists (Tables 1, 2), and the code necessary to control the device (see Github repository https://github.com/lab-instruments).

**Table 1:** Part list for VPS build

Item	Catalog number	Quantity	Manufacturer	Notes
Off-the-shelf components				
Cora Z7: Zynq-7000 Single Core and Dual Core Options for ARM/FPGA SoC	410-370-1	1	Digilent	Main processing board
Pmod SSR: solid state relay electronic switch	410-342	1	Digilent	Relay to control the pinch valve
Pmod AD1: Two 12-bit A/D inputs	410-064	1	Digilent	ADC input
Pmod DA2: two 12-bit D/A outputs	410-113	1	Digilent	DAC output
Power supply components				
ups_pwr2 PCB		1	OSHPARK	https://oshpark.com/shared_projects/CstFkxbF
TERM BLK 2P SIDE ENT 2.54 mm PCB	277-1273-ND	1	Phoenix Contact	Screw terminals for 12VDC
TERM BLK 6P SIDE ENT 2.54 mm PCB	277-1277-ND	1	Phoenix Contact	Screw terminals for GND
DC CONVERTER 5 V 5 W	1951-2747-ND	1	Traco Power	Primary 12VDC to 5VDC converter
Case and external connectors				
USB CIRCULAR CABLE UNSEALED	SAM10681-ND	1	Samtec Inc	For exposing the USB host of the main processing board
ADAPTER USB B RCPT TO USB A RCPT	SAM8637-ND	1	Samtec Inc	For exposing the USB serial Linux terminal of the main processing board
RJ45,C SIZE PANEL SCREW 5E F CON	1754-1125-ND	1	Amphenol LTW	RJ45 Ethernet to the main processing board
CONN PWR JACK 2.5 × 5.5 mm SOLDER	SC1048-ND	1	Switchcraft Inc	12VDC Power input
BOX ALUM GRAY 4.88"L × 7.13"W	HM2530-ND	1	Hammond Manufacturing	Main box
LED PNL IND 7 mm GRN INTERNL	350-2924-ND	1	Dialight	Power indicator light
SWITCH ROCKER SPST 10A 125 V	EG1889-ND	1	E-Switch	Power switch
FUSE HLDR CART 250 V 10A PNL MNT	F3125-ND	1	Littelfuse Inc	Fuse holder for protection
FUSE GLASS 4A 125VAC 5 × 20 mm	F4729-ND	1	Littelfuse Inc	Fuse for protection
DAC/ADC connectorized board				
Ups analog interface PCB		1		https://oshpark.com/shared_projects/eoxPS4Tj
RES SMD 0 Ω JUMPER 1/8 W 0805	311–0.0ARCT-ND	20	Yageo (VA)	Board mount jumpers
CONN SMA JACK R/A 50 Ω PCB	A97593-ND	2	TE Connectivity AMP Connectors	Board mount, right angle SMA connectors
CONN HEADER VERT 6POS 2.54 mm	SAM1029-06-ND	2	Samtec Inc	Pmod to connectorized board connection bus
Pressure sensor	SPD002GAsil	1	Smartec	Pressure transducer sensor
Misc connectors and parts				
1/4 HEX × 1/2 LENGTH	1772-2591-ND	15	RAF Electronic Hardware	Standoffs for board mounting
MACHINE SCREW PAN PHILLIPS 6–32	36-9903-ND	15	Keystone Electronics	Screws for board mounting
Pmod Cable Kit: 6-pin 6ʺ	240-021-6	1	Digilent	Pmod cable to connect main board to Pmod
2 × 6-pin to Dual 6-pin Pmod Splitter Cable	240-110	1	Digilent	Pmod cable to connect main board to Pmod
Pmod Clip: Mechanical Mount for Pmod boards	240-107	1	Digilent	Pmod mount clip
12 V, 3A power supply	240-057	1	Digilent	Main 12VDC supply

**Table 2:** Inline analog-to-digital pressure converter

Item	Catalog number	Quantity	Manufacturer
Platinum Series Digital panel meter	DP8PT-006	1	Omega
Pressure transducer	PX309-005GV	1	Omega
1402 series instrument case	1402 K	1	Hammond

## Methods

### Circuit design

The circuit diagram for the VPS is shown in Fig. 2.

### VPS construction

Off-the-shelf items for the VPS build can be found in Table 1. The majority of the parts are available off the shelf and use with only minor modifications. A custom board was designed to connectorized the ADC and DAC. The board adds a footprint for the pressure transducer for the input to the ADC and two SMA connectors for voltage output from the DAC. The ADC and DAC Pmod boards were modified by removing their normal input/output connectors and soldering directly to the connectorized board. A custom power supply was built to convert from 12 to 5v for the FPGA board and provide 12v power to control the pinch valve. All of the parts were put into a metal project box and the necessary connections are broken out to panel mount connectors including the RJ45 Ethernet connector, USB host, USB device, 12v power input, fuse holder, air inputs/outputs/vents, power switch, power LED indicator, BNC control voltage output and BNC proportional voltage output. The VPS has a total of 4 inlet/outlets (Fig. 1a). One is for the air intake. This air intake is connected to a compressed air tank with an analog pressure control valve used to regulate air input to the VPS (Fig. 3). A second outlet is an exhaust valve for quick reset of the system at the end of a trial. The third and fourth valves are outlets for the air stimulus to the mouse catheter.

### Coding and software

The code for all parts of the project is available under the Github repository https://github.com/lab-instruments. The code is broken into the platform firmware and the desktop GUI. All code is released under the MIT license.

The platform firmware is located under the repository located at https://github.com/lab-instruments/ups. This repository provides the necessary source code and scripts to completely build from source the embedded Linux, u-boot and FPGA binary. The repository also includes the Python servers and embedded platform startup scripts. To build the platform code, a Linux (tested on Ubuntu) PC, Xilinx Vivado 2018.2 and various packages are required. The script located at ups/init/init_box.sh can setup and install all required software. The build (u-boot, embedded Linux and the FPGA code) is fully scripted and automated including TCL based FPGA development tool automation and SD card creation and writing (for the FPGA board). The FPGA code is written using standard System Verilog and industry standard coding methods. The embedded Linux was built using the Buildroot tool and a custom configuration script.

The GUI software with installation instructions is located under the repository located at https://github.com/lab-instruments/ups_python. The GUI is written in pure python and requires Python3. To run the GUI, you need Python3 and various other libraries installed. These libraries include (1) “PyQt5” (GUI libraries; https://pypi.org/project/PyQt5/), (2) “netifaces” (library to examine hardware network interfaces; https://pypi.org/project/netifaces/), and (3) “requests” (an HTTP interrogation library; https://pypi.org/project/requests/). A setup script called ups_python/python/setup_venv.sh sets up a Python virtual environment with all the required dependencies. The ups_python/python/gui.sh utilizes the virtual environment setup by setup_venv.sh and launches the GUI. The GUI is cross-platform and should run on any PC or Mac that supports Python3 and PyQT5.

### Animal experimentation

Animal usage was approved by the University of Texas at Dallas Institutional Animal Care and Use Committee. For the primary demonstration of the unit, one female C57Bl/6J mouse (adult 12 weeks old) was used in this study. We also included data from three additional mice (female C57Bl/6J adult 12 weeks old) that were used in real-world experiments as described below to compare the pressure measured read by the VPS and the pressure from our digital-to-analog converter. The visceromotor response (VMR) is a spinobulbospinal reflex and quantitative measure of these pain-like responses. VMRs are obtained by measuring the electrical activity (EMG) of the external oblique abdominal muscle during bladder distension; this EMG is a reliable, reproducible measure of nociception that is exacerbated by bladder sensitization and reduced following analgesic administration [20]. In these experiments, UBD was performed as previously reported [6, 7, 19] (Fig. 3). Briefly, mice were anesthetized with isoflurane in an induction chamber then transferred to a nose cone administering 2% isoflurane (vaporized with 100% O_2_). An incision was made in the lower skin of the abdomen and two silver wires were implanted in the left abdominal muscle. A third grounding wire was laced through the skin overlying the chest cavity. Lastly, a lubricated 24 gauge, 14 mm angiocatheter was inserted in the bladder via the urethra.

Following surgical completion, isoflurane was lowered to 1.5% for 10 min. Isoflurane was then gradually lowered by an additional 0.125% every 10 min until animals responded to noxious toe pinch but were not vocalizing or ambulating (typically 0.8–0.875%). Once at a stable isoflurane level, animals’ bladders were distended three times with 30 mmHg compressed air. A y-splitter in the VPS-to-catheter tubing was used to independently measure pressure during stimuli for comparison to the pressure reader in the VPS. Two different devices were used to independently measure the pressure. This included a commercial analog sphygmometer (Criterion Model 85G, Prestige Medical with a manufacturer set tolerance of ± 3 mmHg) and a custom inline analog-to-digital pressure converter (Table 2). The converter consisted of a pressure transducer (Omega Manufacturing, Model PX309-005GV) and a calibrated pressure reader (Omega Manufacturing, Model DP8PT-006) with a BNC output to an ADC input on a Micro1401-4 (Cambridge Electronic Design (CED)).

Two BNC outputs from the VPS were connected to ADC inputs on the Micro1401-4. Each distension lasted 20 s with a 1 min intertrial interval (ITI). The VMR signal was relayed in real-time using a preamplifier (World Precision Instruments ISO-80) connected to the Micro1401-4. The Micro1401-4 was connected to a PC equipped with Spike2 data acquisition software (Version 8, CED).

#### Demonstration data

Demonstration mouse was given three 20 s distention trials targeted at 30 mmHg with 1 min in between trials. The actual air pressure is controlled with a dial and analog sphygmometer. Thus, while 30 mmHg is the target pressure, the actual pressure (measured in a trial from the VPS and secondary analog-to-digital pressure converter) is always slightly off from the target (as seen in Table 3). 30 mmHg is thought to be relatively innocuous while still eliciting a VMR (similar to filling of the bladder after drinking a cup of coffee). Raw data was exported individually from each trial starting ~ 0.5–0.75 s prior to the pre-distention signal marker to plot raw data (Fig. 4). When plotting these data, *x*-axis was manually adjusted so that all three trial pre-distention marker signals matched. Pressure read on Spike2 by the VPS and the digital-to-analog converter were analyzed in Spike2 to determine the average pressure across each trial. In addition, all data from the experiment including background data between trials was exported from Spike2 for analysis of VMR. VMR signal was subtracted from background EMG activity, rectified, and integrated over the distension periods, as previously described [6, 19], using a custom script written in MatLab (MathWorks). To normalize, all distension VMRs were divided by the smallest pre-distension VMR for the individual experiment.

**Table 3:** Comparison of pressure outputs for demonstration trials

Trial #	Target pressure (mmHg)	Analog Sphyg. (mmHg)	VPS (mean ± SD) (mmHg)	Analog-to-digital converter (mean ± SD) (mmHg)	MatLab avg pressure from VPS signal	MatLab VMR signal (V * s)
1	30	31 ± 3	32.92 ± 0.53	32.15 ± 0.70	32.83	1.33
2	30	31 ± 3	32.98 ± 0.57	32.16 ± 0.78	32.95	1.59
3	30	32 ± 3	32.34 ± 0.84	31.17 ± 1.16	32.33	1.22

Data show pressure and calculated VMR for 3 trials (20 s of actual stimulus; total trial length 40 s; intertrial interval of 1 min) between the VPS, the inline analog-to-digital pressure converter and a commercial analog sphygmometer. The Matlab avg pressure was calculated from VPS signal and the Matlab VMR signal was calculated from the normalized EMG signal

#### Real-world experimentation

After a successful demonstration of the device in a wild-type mouse, we sought to utilize the VPS in our real-world laboratory experiments. Over the course of a 2-month period, we used the system described here for 18 mice. The total number of trials completed with the VPS (and other system components described above) was 1200. Here, we have analyzed pressure data from three of these mice to further validate the VPS system. These mice received alternating 20 s pressure trials of 30 and 60 mmHg with 1 min in between trials. Targeted 60 mmHg pressure is thought to be noxious (similar to a full bladder). We report 20 total trials per animal (10 at 30 mmHg and 10 at 60 mmHg). Pressure data were processed and analyzed as above in Spike 2 for the demonstration trials. 30 mmHg and 60 mmHg data were separately analyzed using SPSS (v 1.0.0.1406) and plotted with Prism (v 8.0) to determine intra-rater reliability using an intra-class correlation coefficient (ICC). See Additional file 1 for the raw data from these trials.

## Supplementary Information

**Additional file 1.** This is the raw data used for Figure 4 and 5.

## Data Availability

Raw data from three UBD-VMR trials is included in Additional file 1. Code for VPS and VMR-UBD analysis script (MatLab) can be found at https://github.com/lab-instruments.

## Abbreviations

EMG: Electromyogram
GUI: Graphic user interface
ICC: Intraclass correlation coefficient (ICC)
UBD VMR: Urinary bladder distention visceromotor response
VPS: Visceral pain stimulator
